# Dynamic Metabolism in Immune Response

**Published:** 2016-04-29

**Authors:** Mazen Al-Hommrani, Paramita Chakraborty, Shilpak Chatterjee, Shikhar Mehrotra

**Affiliations:** * Departments of Surgery, Microbiology & Immunology, Medical University of South Carolina, Charleston, SC 29425, USA

## Abstract

Cell, the basic unit of life depends for its survival on nutrients and thereby energy to perform its physiological function. Cells of lymphoid and myeloid origin are key in evoking an immune response against “self” or “non-self” antigens. The thymus derived lymphoid cells called T cells are a heterogenous group with distinct phenotypic and molecular signatures that have been shown to respond against an infection (bacterial, viral, protozoan) or cancer. Recent studies have unearthed the key differences in energy metabolism between the various T cell subsets, natural killer cells, dendritic cells, macrophages and myeloid derived suppressor cells. While a number of groups are dwelling into the nuances of the metabolism and its role in immune response at various strata, this review focuses on dynamic state of metabolism that is operational within various cellular compartments that interact to mount an effective immune response to alleviate disease state.

## INTRODUCTION

Cells depend on nutrients available in their extracellular environment to support the biochemical processes that are required for cell growth and proliferation. The cells responsible for mounting adaptive immunity in response to pathogens or cancers require a set of complex but coordinated signals to drive their activation, proliferation, and differentiation. It is increasingly clear that all cell types have cellular metabolism coupled with various stages in their life-span to meet the energetic requirements for survival. A comprehensive understanding about the role of metabolism in cellular function is therefore important for developing novel therapeutic approaches to treat various diseases or cancer. Here, we discuss briefly recent studies that highlight the role of metabolic pathways or metabolites in the function of both lymphoid and myeloid cells.

### Immunometabolism of Lymphoid Cells

#### T cell

The activation of the naïve T cell either through T cell receptor (TCR) engagement (or) by a mitogen leads to numerous changes in its proliferation/expansion and renders the activated T cells with distinct phenotype and function [1]. T cell activation also leads to rapid shifts in cell metabolism to co-opt the bioenergetic needs of a rapidly proliferating T cell [2]. Quiescent T cells are in continuous need for cellular energy provided by adenosine triphosphate (ATP) consumption for their migration and persistent cytoskeletal rearrangement; therefore they rely preferentially on the growth-promoting pathways as oxidation of pyruvate, fatty acid and glutamine [2]. Early study by Rathmell *et al.* showed that in the absence of extrinsic signals, nutrient utilization by lymphocytes is insufficient to maintain either cell size or viability [3]. Their study demonstrated that after TCR engagement was lost, lymphocytes rapidly down regulated the glucose transporter, Glut1 along with reduced mitochondrial potential and cellular ATP. Another study from Craig Thompson’s group showed that second signal in form of co-stimulation leads to bioenergetics modulation that results in a decision on anergic *vs.* effector T cell response [4]. Further, work by Jonathan Powell’s group elegantly showed that anergic T cells are in fact metabolically anergic as well [5]. An important observation from Thomas Gajewski’s group showed that effector cytokine secretion by activated T cells is dependent on availability of glucose, and inhibiting glycolytic pathway using 2-deoxyglucose (2-DG) results in loosing cytokine secretion [6]. Thus, these pioneering studies firmly established that glucose metabolism in lymphocytes is a regulated process that effects on immune cell function and survival [7]. Activation of T cells not only results in increase in Glut1 expression and surface localization, but if glucose uptake is limited, glycolytic flux decreases to a level that no longer sustains viability, and proapoptotic Bcl2 family members become activated, promoting cell death [7].

### T cell subsets and metabolism

Given the heterogenous phenotype of both CD4^+^ T helper (Th) and CD8^+^ T cytotoxic (Tc) cells that also differentiate to distinct lineages based on effector cytokine secreting signature (*i.e.*Th1/Tc, Th2/Tc2, Th9/Tc9, Th17/Tc17, Treg’s), it is important to determine if all these T cell subsets follow similar or unique metabolic signature. Seminal studies from Rathmell’s group showed that Th1,Th2, and Th17 cells strongly engage glycolysis, whereas tolerance inducing regulatory T cells (Treg’s) depend more on the oxidative phosphorylation to fulfill their bioenergetics demands [8]. Similar to CD4^+^ T cell, CD8^+^ T cells that differentiate to effector cytolytic T cells following activation preferentially use glycolysis as their major bioenergetic pathway [9], whereas, the small percentage of CD8 T cells which persist as memory cells after contraction of the effector phase mostly rely on the oxidative phosphorylation for energy [10]. Primarily, glycolysis is an anaerobic metabolic pathway happening in the cytosol. It degrades glucose to yield pyruvate and other precursors needed for cellular anabolism. For every molecule of glucose metabolized through the glycolytic pathway two molecules ATP are produced. Activated effector T cells convert most of the pyruvate, a downstream product of glycolysis, into lactate instead of Acetyl-CoA that can be oxidized in mitochondria [2]. Even though glycolysis has low yield of ATP, it is considered to be a preferable pathway over oxidative phosphorylation, which has high ATP output, for activated effector T cells. This switch into glycolysis by stimulated T cells is important for NADPH production and nucleotide synthesis which are needed by proliferating T cells. Recently, a glycolytic enzyme glyceraldehyde 3-phosphate dehydrogenase (GAPDH) has been shown as a post-transcriptional inhibitor of interferon gamma mRNA and once GAPDH engages in glycolysis it releases IFNγ mRNA leading to IFNγ production [11]. Even though glycolysis pathway is important for activated effector T cells, mitochondrial pathway is not completely inhibited. Instead, glutamine oxidation by mitochondria is enhanced in activated T cells to replenish tricarboxylic acid cycle (TCA) and produce reactive oxygen species (ROS), which has been proven to be important for activation of T cells and signaling of interleukin 2 production [12]. Oxidative phosphorylation (OXPHOS) is the major pathway of producing ATP. Glucose, glutamine and fatty acid are oxidized in mitochondria via TCA cycle to generate reducing equivalents, such as nicotinamide adenine dinucleotide (NADH) and flavin adenine dinucleotide (FADH2). These molecules then feed the OXPHOS pathway by donating electron to electron transport chain (ETC). OXPHOS pathway are favored by regulatory T cells (Treg’s) and memory T cells for their development and long-term survival [13]. Also, memory T cells maintain greater mitochondrial mass than effector T cells which indicate their dependence on OXPHOS pathway [14]. Enhancement of fatty acid oxidation promotes the development of memory CD8+ T cells after immunization [15–19]. Thus, the changes in metabolism between glycolysis and OXPHOS pathway are mandatory to maintain T cell function and efficient progression from naïve phenotype to effector and memory phenotype.

It is also important to understand the underlying mechanisms of adaptation of distinct metabolic programming in effector *vs.* Treg (or memory T) cells following encountering immunological signals which drive them into different functional subsets. Recent studies have shown that effector T cells express high surface levels of the glucose transporter Glut1 that makes them highly glycolytic [9]. In contrast, Treg’s express low levels of Glut1 and have high lipid oxidation rates [8]. It has been shown that blocking glycolysis inhibits Th17 development while promoting Treg cell generation [20]. Further, it has been also shown that the effector T cells exhibit the metabolic phenotype that is not fixed [21]. However, the state is changeable or dynamic between the OXPHOS and Glycolysis. Upon activation, mitogen-activated T cells have been documented to switch to glycolysis, less sufficient pathway of energy production, to support their biosynthesis processes [8]. Some of the activated T cells survive to form long lived memory T cells and switch to β-oxidation of fatty acid [22]. Similarly, regulatory T cells have shown high lipid oxidation in vitro [8]. The fate of an activated T cells depend on many factors such as the strength of TCR signaling, costimulatory molecules and cellular microenvironment. Cellular microenvironment is represented by nutrition and oxygen level surrounding activated T cells. These factors highly affect mammalian target of rapamycin signaling pathway (mTOR). Suboptimal signaling of mTOR pathway during starvation results in generation of Tregs and inhibition of effector T cells [23]. Rapamycin, inhibitor of mTOR pathway, has similar effect, it inhibits T cell proliferation and selectively increase Treg generation [24]. Glut, glucose uptake receptor, transgenic T cells have shown more proliferation and cytokine production; Treg’s, in contrast, had normal response [8]. Fatty acid oxidation has equal importance for Treg’s and memory T cells as glucose uptake for activated effector T cells. It has been found that inhibition of lipid oxidation through CPT1a, carnitine palmitoyltransferase 1A, compromises Treg development, whereas increase provision of lipid either suppresses effector T cells or enhances Treg’s proliferation [8]. Unlike Treg’s which use extracellular lipid to produce phospholipid used in the formation of cellular membrane, Th17 cells use endogenous fatty acid synthesis for the same purpose [25]. Each T cell subset has its own metabolic signature that can be targeted by developing a pharmacological drug that can help either in enhancement or suppression of T cell function. Therefore, it is likely that transition from one metabolism pathway to another dictate T cell functions and shapes their different subsets. This phenomenon has been employed by tumor cells through high consumption of glucose, an important substrate for effector T cells, in their microenvironment. Also, tumor cells bind programmed death molecule-1 (PD-1) on exhausted T cells which target downstream signaling leading to a decrease in glucose metabolism.

With regards to the role of metabolism in pathogenic T cells, it has been shown that alloreactivity T cells in graft versus host disease (GVHD) enhance both glycolysis and OXPHOS pathway with low glutathione and high reactive oxygen species (ROS) production [26]. Increasing ROS production by using Bz-42, an inhibitor of mitochondrial F1F0-ATPase, selectively induced apoptosis in alloreactivity T cells but not resting T cells or proliferating bone marrow cells [27]. Rapamycin has long been used as a potent immunosuppressive therapy in transplantation by targeting mTOR pathway [28], a central regulator of T cell activation. TCR-dependent signaling of mTOR pathway leads to Glut1 increase, and subsequently glucose uptake associated with effector T cell phenotype. Also, mTOR downstream signaling of HIF1α which further induces glycolytic pathway selectively enhances Th17 development over Treg by activating transcription factor RORγ [20]. Likewise, AMP-activated protein kinase (AMPK) activation, which enhances fatty acid oxidation (FAO) also alters this balance in favor of Treg cells [29]. HIF1α deficient mice have diminished Th17 development but enhanced Treg cell differentiation and protected mice from autoimmune neuroinflammation [20]. Furthermore, inhibition of acetyl-CoA Carboxylase 1(ACC1) used by Th17 for endogenous fatty acid synthesis attenuates Th17 mediated autoimmune disease.

The inhibition of pyruvate dehydrogenase kinase (PDK1) enzyme, which is upregulated during T cell activation to prevent pyruvate from being oxidized in mitochondria and rather forming lactate, has led to inhibition of collagen-induced arthritis in female mice [30]. Moreover, PDK1 inhibition has an effect on human and mouse asthma model by inhibiting lactate production, proliferation of T cells and production of IL17 a**n**d IFNg on other hand, it stimulates the production of IL −10 and the induction of Foxp3 [31]. During switching from effector T cells toward memory T cells, memory CD8 T cells switch to fatty acid oxidation and down regulate glycolysis. High glycolysis uptake in CD8 T cells has been shown to be associated with compromise in the generation of long-lived memory cells by driving T cells toward a terminally differentiated state [32]. In the same study, it has been reported that Pmel (pre-melanosome protein) transgenic CD8 T cells co-cultured with 2DG, glycolysis pathway inhibitor, have better antitumor effect in adoptive T cell therapy, indicated by smaller tumor size and longer survival time [32]. To obtain a robust long lived CD8 memory T cells many studies showed that enhancement of fatty acid oxidation by treating CD8 T cells with either metformin, activator of AMPK pathway, or inhibitor of mTOR pathway has led to the development of CD8 memory T cell after immunization [15–19]. Thus, regulating T cell metabolism is a promising target for immunotherapy to maintain the balance of Treg and Teff cells and enhance CD8 memory T cells that are important in tumor adoptive T cell therapy.

Further, the cytokines that help expanding T cells have been shown to play a role in modulating T cell metabolism. Using the cytokines interleukin (IL)15 and IL-2, it was shown by Pearce et al that memory T cells generated with IL15 exhibit enhances spare respiratory capacity, as compared to the effector T cells generated with IL2 [33]. This study showed that IL15, a cytokine critical for CD8+ memory T cells, regulated spare respiratory capacity and oxidative metabolism by promoting mitochondrial biogenesis and expression of carnitine palmitoyl transferase (CPT1a), a metabolic enzyme that controls the rate-limiting step of mitochondrial fatty acid oxidation (FAO). These results established how cytokines control the bioenergetic stability of memory T cells by regulating mitochondrial metabolism. It has also been recently shown that IL7, that plays an important role in homoeostatic proliferation and differentiation, induces expression of the glycerol channel aquaporin 9 (AQP9) in virus-specific memory CD8+ T cells, but not naive cells, and that AQP9 is vitally required for their long-term survival [33a]. AQP9 deficiency impairs glycerol import into memory CD8+ T cells for fatty acid esterification and triglyceride (TAG) synthesis and storage. These defects can be rescued by ectopic expression of TAG synthases, which restores lipid stores and memory T cell survival. This study uncovers the metabolic mechanisms by which IL-7 tailors the metabolism of memory T cells to promote their longevity and fast response to rechallenge. Therefore, strategies to modulate metabolic pathway of T cell subsets could result in growth enhancement/deterioration and hyper-functionality or dysfunctionality with implications in autoimmune diseases and cancer.

#### B cells

B cells originate in the bone marrow from hematopoietic stem cells which give rise into multipotent progenitor (MPP) cells, then common lymphoid progenitor (CLP) cells. CLP differentiates toward natural killer cells, T cells and B cells. Unlike NK cells and T cells which mediate cellular immunity, B cell main function is mediating humoral immunity. B cells migrate from bone marrow to the spleen as immature B cells [34]. Once they are in the spleen, they differentiate toward follicular B cells or marginal zone B cells. There are many types of B cells, most common one is follicular B cell (FB cell) that if not circulating through the body, reside in the follicles of secondary lymphoid organ [35]. Following FB cell is marginal B cell that resides in the marginal zone of lymph node and form the first defense of pathogen encountered in the secondary lymphoid organ. After activation, B cells mature toward antibody secreting plasmablast which later form long lived plasma cells residing in the bone marrow. Recently, a new class of B cell is discovered called memory B cell that is rapidly reactivated to produce antibodies. While the understanding of B cell metabolism is not as abundant as T cells, here we cover the recent studies that have demonstrated the metabolic regulation of B cell responses.

The metabolic re-programming of activated immune cells now is gaining more attention and dramatically regulates immune cell function. As in T cells, AKT/PI3K pathway plays a key role in upregulation of Glut1 receptor to enhance glucose uptake upon antigen stimulation in B cells and blocking this pathway prevents BCR mediated growth [36]. Glycolysis plays a key role in T cell proliferation, activation and cytokine production [37]. Similarly, B cell engaged glycolysis and formed lactate when it was measured at 24hrs and 48hrs [36], showing a preference for glycolysis pathway over OXPHOS. The FcγRIIB is a potent inhibitory co-receptor that blocks BCR signaling in response to immune complexes and, as such, plays a decisive role in regulating Ab responses [38]. It is noteworthy that co-ligation of the BCR and FcγRIIB has led to almost 82% reduction in glucose uptake when compared with B cells stimulated via the BCR alone [36]. This shows that glycolysis is a key player in B cell activation. While, activated T cells and tumor cells show predominant transition into glycolysis with low OCR/ECAR ratio [33, 39–41], the OCR/ECAR ratio was found to remain unchanged in B cells after stimulation with LPS [42]. Consequently, B cells are unique and increase both glycolytic and mitochondrial metabolic activity in a balanced fashion after LPS stimulation [43]. Unlike Th17 cells, B cells do not require HIF1α to induce glycolysis- as B cells from HIF1α deficient mouse induced glycolysis as comparable to wild type mouse after LPS activation [20,43]. In contrast to HIF1α, c-Myc, a transcription factor important for cell proliferation, has been shown to enhance glycolysis gene expression and glutamine metabolism in both activated T cell and B cell [43,44]. It was demonstrated that Myc-deficient B cell failed to increase ECAR rate as well as OCR [43]. In the same study, lipid oxidation decreased whereas pyruvate oxidation increased after LPS activation [43]. Compromising aerobic glycolysis pathway by either treating B cell with glucose inhibitor, pyruvate dehydrogenase kinase inhibitor or down-regulating Glut1 receptor leads to suppression of Ab production. This indicates that Ab production by B cells is controlled by glucose substrate as that of IFNγ by T cells.

Cytokines play a role in the activation and programming of immune cells. B cell activating factor (BAFF), secreted by myeloid cells, maintains B cell survival and differentiation [45]. It acts as a co-stimulatory signal in B cell activation. Chronic exposure to BAFF leads to increase metabolic capacity of B cells. Glucose uptake as well as ECAR rate increased after 6hrs activation with LPS in BAFF transgenic B cells [43]. BAFF signals B cell through PI3K/AKT pathway [46], and consequently leads to increase glucose uptake. Expose B cells to elevated levels of BAFF leads to a spontaneous SLE-like disease in mice [47]. As a result, BAFF seems a promising target in autoimmune disease.

In the intestine, naive B cell residing in the Payer’s patch differentiate into IgA producing plasma cells that migrate to intestinal Lamina propria (iLP). Kunisawa et al. has shown that naïve B cells depend on TCA cycle and Vitamin B, enzymatic cofactor for TCA cycle’s enzymes, for their energy production [48]. Inhibition of TCA cycle or deletion of Vitamin B1 from the nutrient result in a low number of naïve B cells as well as prevent their proliferation [49]. On the other hand, IgA plasma cells (PCs) in the iLP obtain energy (ATP) from both glycolysis and TCA cycle. PCs are not intensely affected by inhibiting OXPHOS pathway or deletion of vitamin B1. This study shows that different B cell subsets have a distinct metabolic pathway, which is poorly understood and needs further investigation.

#### Natural Killer (NK) Cell

NK Cells have been known to be the first line of defense against the invading non-self or self-antigens [50]. Recent studies have now started to dissect the role of metabolism in NK cell mediated effector and memory responses [51], which we briefly discuss here. It has been shown that mTORC1-dependent metabolic reprogramming is a prerequisite for NK cell effector function [52]. This study showed that NK cells undergo dramatic metabolic reprogramming upon activation, up-regulating rates of glucose uptake and glycolysis, and that mTORC1 activity is essential for attaining this elevated glycolytic state. Directly limiting the rate of glycolysis is sufficient to inhibit IFNγ production and granzyme B expression. Similarly, it is shown that the metabolic checkpoint kinase mTOR was activated and boost bioenergetic metabolism after exposure of NK cells to high concentrations of IL15, whereas low doses of IL15 triggered only phosphorylation of the transcription factor STAT5 [53]. However, another study has shown that the degree of activation of NK cells regulate it’s metabolic commitment [54]. This study investigated the metabolic requirements for production of IFNγ by freshly isolated NK cells, and showed significant differences in the metabolic requirements of murine NK cell IFNγ production depending upon the activation signal [54]. A striking result was that stimulation of NK cell IFNγ production was independent of glycolysis or mitochondrial oxidative phosphorylation when cells were activated with IL12 plus IL18. However, stimulation via activating NK receptors required glucose-driven oxidative phosphorylation. Importantly, prolonged treatment with high-dose, but not low-dose, IL15 eliminated the metabolic requirement for receptor stimulation. Thus, these handful studies on NK cell metabolism indicate that with a complex network of activation and inhibitory receptors there may be an important role of metabolic regulators that control a robust NK cell activation, exhaustion or memory response.

### Immunometabolism in Myeloid Cells

In addition to lymphoid cells, a good bit of recent literature highlights the importance of understanding metabolism in the myeloid cells as dendritic cells, macrophages or myeloid derived suppressor cells. We discuss here briefly about the role of metabolism in shaping the phenotype and function of myeloid cells.

#### Dendritic Cell

Dendritic cells (DCs) have been known as the professional antigen presenting cells that respond to pathogens or other danger signals and initiate innate and adaptive immune responses. An increased understanding of DC metabolism under different disease states such as infection or tumor is important to understand how the innate effectors get activated or programmed to respond and control such pathological conditions. A recent study from the Edward Pearce’s group showed that stimulation of TLR induces a metabolic transition in resting immature DC, characterized by a conversion from mitochondrial β-oxidation of lipid and OXPHOS to aerobic glycolysis [55]. It was a surprising finding that DCs that do not undergo robust proliferation (as compared to tumor cells or T cells) also depend upon glucose availability for optimal maturation (as seen by upregulation of CD40, CD80, and CD86) and survival. It is thus likely that DCs in tumor microenvironment are rendered dysfunctional in terms of antigen presentation or secreting immunogenic cytokines as they compete for glucose substrate with the highly glycolytic tumor cells. Similar to cancer cells and effector T cells, PI3K/AKT pathway has been shown to play a key role in controlling metabolic transition to glycolysis in TLR-stimulated DC [55]. AKT promotes glycolysis in DC in part by increasing the expression of Glut-1 and likely activates downstream mTOR pathway. However, some reports have shown that inhibition of mTOR by rapamycin in murine GM-CSF-driven DC and human myeloid DC prolong the lifespan, promote expression of co-stimulatory molecules and cytokines, and enhances DC immunogenicity [56]. It is likely that the persistent stimulation signals in form of pro-inflammatory cytokines or duration of Toll like-receptor (TLR) engagement may have a role in level of mTORC1 or mTORC2 involvement leading to differences in DC metabolic state and may account for variable observations.

DCs that are generated from a common myeloid progenitor in the bone marrow can also be differentiated toward different DC subsets. To date, there are four identified subsets of DCs: classical DCs, monocyte derived DCs, plasmacytoid dendritic cells (pDCs) and Langerhans cells [57]. They are well-known as mediators of innate and adaptive immune response rendering them critical for immunotherapy. As a result, large focus has been recently shed on the metabolism of DCs subtypes. In vitro differentiation of monocyte derived DCs and in vivo development of DCs require fatty acid synthesis [58]. On the contrary, fatty acid synthesis blockade enhanced bone marrow derived DCs immunogenicity by showing increase antigen uptake, pro-inflammatory cytokine production, and priming of Ag-restricted CD4+ and CD8+ T cells [58]. These results indicate that DCs function is tethered to their metabolism that can be a promising target for immunotherapy. Peroxisome proliferator receptor-γ (PPARγ), mediator of fatty acid metabolism, along with mitochondrial biogenesis regulator, PPARγ co-activator 1 α is shown to be increased in in vitro differentiated monocyte-derived DCs [59,60]. Also, inhibiting electron transport chain (ETC) has led to a prevention of DCs differentiation from monocyte which can be reflected by the low expression of CD1a differentiation marker [61]. The development of cDCs and pDCs from committed dendritic cells progenitor (CDP) depends on cytokine signaling from FMS-like tyrosine kinase 3 (Flt3) receptor expressed by CDP [62]. Also, providing CD8+ DCs and their corresponding CD103+ tissue DCs with Flt3 cytokine ligand (Flt3L) leads to increase in their expansion [63]. Rapamycin mediated inhibitor of mTORC1 compromises Flt3 ligand signaling leading to prevention of pDCs and cDCs growth in vitro as well as in vivo [64, 65]. Moreover, phosphate and tensin homologue (PTEN), a negative regulator of mTOR pathway, deletion enhanced in vivo expansion of cDCs and pDCS, an effect that can be abrogated by rapamycin [65]. MYC, a transcription factor, is a downstream signaling of mTOR pathway and responsible for gene expression of glycolytic protein [66]. It has been shown that Myc paralogue, L-Myc, deficiency reduced the number of migratory CD103+ DCs, beside splenic CD8+ and CD8- DCs [67]. In addition to the reduction of number also antigen priming was lost in CD8+ DCs and pDCs [67]. One of the genes that are targeted by L-Myc is NADH dehydrogenase (complex I) that impacts the energy metabolism of DCs [67]. Resting bone marrow derived dendritic cells (BMDCs) use both fatty acid oxidation in mitochondria and glycolysis when they are induced by granulocyte macrophage colony stimulating factor (GM-CSF) [55]. However, it is not quite clear if cDCs and pDCs oxidize fatty acid as well. It is now clear that monocyte derived DCs and cDCs increase glucose flux at early stage of activation [55]. Hypoxia faced in inflammatory state along with Toll like-receptor (TLR) activation has been shown to drive DCs shifting metabolism toward glycolysis via activation of HIF1α [68]. Thus, dendritic cell metabolism seems to undergo dynamic change to accommodate their microenvironment and execute their function. Using 2-DG, inhibitor of glycolysis pathway, render DCs inactive [69]. AMP-activated protein kinase (AMPK), which mediate inhibition of mTOR pathway and enhance OXPHOS pathway [70,71], suppresses TLR-induced glucose consumption and consequently activation of DCs [72]. Even though, it seems that DCs follow Warburg effect by increasing glucose uptake after activation, it has been revealed that rapid incorporation of glucose-derived carbon into TCA cycle enhanced at early stage of activation [69]. The reason of glucose oxidation is assumed to facilitate a transient increase in spare respiratory capacity [73] and the use of TCA intermediate, citrate, for fatty acid synthesis [69]. After 12 hour of BMDCs activation, their metabolism almost entirely switches toward glycolysis [55,74]. This is attributed to the production of nitrogen oxide (NO) from inflammatory DCs which can block ETC. This might be one of the reasons behind switching toward Warburg metabolism in the absence of increase proliferation [74]. Interestingly, mTOR inhibition associated with iNOS down regulation leads to increase DCs lifespan and maintain their mitochondrial function [75,76]. The role of fatty acid synthesis in activated DCs is not clear since these cells do not proliferate after activation. Nonetheless, the necessity to increase the mass of Golgi and endoplasmic reticulum (ER) as a result of increase protein synthesis could explain the demand for fatty acid synthesis [69]. *De novo* fatty acid synthesis and lipid content is correlated with immunogenicity of liver DCs [77]. On the contrary, high lipid content decrease their immune priming function in tumor microenvironment [78].

Eventually, many factors affect the metabolism of DCs such as oxygen and nutrients availability and microenvironment where DCs reside in, i.e. tumor or sites of inflammation. However, it is poorly understood why DCs adopt fatty acid synthesis at resting state and whether different DCs subsets have distinct metabolic requirements.

#### Macrophages and Myeloid-derived suppressor cells

Myeloid-derived suppressor cells (MDSC) are one of the major components of the immunosuppressive network responsible for immune cell tolerance in cancer [79–84]. Polarized MDSC lineages can be distinguished as M1 and M2 cells. M2 can be induced by interleukin (IL)-4 or IL-1, and produce arginase 1 and anti-inflammatory cytokines, eventually converging to facilitate tumorgenesis [83–87]. In marked contrast, M1 could be induced by lipopolysaccharide (LPS) or/and IFN-γ and produce inducible nitric oxide synthase (iNOS), nitric oxide (NO), and pro-inflammatory cytokines, leading to their antitumor effects [79, 80,85]. A recent study has determined the mechanisms that underlie differentiation of MDSCs into M1 or M2 myeloid lineage and their effect on cancer pathophysiology. They observed that glycolytic activation through the SIRT1-mTOR/HIF -1α pathway was required for differentiation to the M1 phenotype [88]. This implies that SIRT1 is a key factor in the regulation of MDSC differentiation into M1 and M2 phenotypes through hypoxia-inducible factor-1α (HIF -1α) –induced glycolytic metabolic reprogramming and has an impact on MDSC functions in both immune suppression and promotion of tumor progression. It was also established by another study that functional polarization of tumor-associated macrophages is mediated by tumor-derived lactic acid that is regulated by HIF1α [89]. In addition, the lactate-induced expression of arginase-1 by macrophages was shown to play an important role in tumor growth. Recently, another study showed that tumor-infiltrating MDSC (T-MDSC) increased fatty acid uptake and activated fatty acid oxidation (FAO) [90]. This was accompanied by an increased mitochondrial mass, up regulation of key FAO enzymes, and increased oxygen consumption rate. Pharmacologic inhibition of FAO blocked immune inhibitory pathways and functions in T-MDSC and decreased their production of inhibitory cytokines. FAO inhibition alone significantly delayed tumor growth in a T-cell-dependent manner and enhanced the antitumor effect of adoptive T-cell therapy.

### Metabolic Interactions, Redundancy and Immune outcome

The redundancy or overlap of pathways involved in generating energy for the cellular functions still exists. One of the previous study also shows that mitochondrial ATP is essential for the rapid induction of glycolysis in response to activation and the initiation of proliferation of both naïve and memory T cells [10]. While this study reconfirmed the finding that similar to CD4+T cells, CD8+ memory T cells also depend on fatty acid oxidation for bioenergetics requirements, they also demonstrate that dissociation of the glycolysis enzyme hexokinase (HK) from mitochondria impairs proliferation and blocks the rapid induction of glycolysis upon T-cell receptor stimulation in memory T cells. It must be noted that hexokinase-mitochondrial interaction has been shown to regulate glucose metabolism differentially in adult and neonatal cardiac myocytes [91]. In this adult myocyte model it was shown that while over expression of HKI, but not HKII, increased glycolytic activity – demonstrating that differential interactions of HKI and HKII with mitochondria underlie the different metabolic profiles. Further, the role of Hexokinase-Mitochondria Interaction has been established in Akt mediated inhibition of apoptosis [92]. This study showed that targeted disruption of mitochondria-hexokinase (HK) interaction or exposure to pro--apoptotic stimuli that promote rapid dissociation of hexokinase from mitochondria potently induce cytochrome c release and apoptosis, even in the absence of Bax and Bak. It is intriguing that despite the widely appreciated anti-apoptotic activity of Akt that is coupled, at least in part, to its effects on cellular metabolism-a study also showed that pharmacologic inhibition of Akt enables expansion of potent tumor-specific lymphocytes with the transcriptional, metabolic, and functional properties characteristic of memory T cells [93]. While glycolytic pathway enzymes (as HKII or GAPDH) or metabolites may be important for effector or memory T cell energetics, it is to remember that end product of lactic acid that exits from the cell in the microenvironment causes an unfavorable environment for normal cells. An earlier study has shown that acidic pH results in T cell dysfunctionality and death [94]. Overall, it is acceptable fact that the effector cytokine function of the lymphoid cells is primarily dependent upon the glycolytic pathway. The translation of key effector cell cytokine IFNγ has been shown to be regulated by the sustained glycolysis, through glyceraldehyde 3-phosphae dehydrogenase (GAPDH) with the 3’ untranslated region (UTR) of the IFNγ mRNA [95]. In addition, inhibition of glycolysis or the use of alternative oxidative fuel as galactose resulted in increased expression of immune inhibitiory receptor PD1, which has been extensively shown to inhibit T cell response [95]. However, since oxidative phosphorylation and spare respiratory capacity (SRC) has been shown to be important for generation of T cell memory response [40], it remains to be established if galactose cultured T cells that have higher oxidative metabolism and SRC will be able to treat tumors.

### Targeting Metabolism

Dampening the immune response using metabolic targets has already been tried in autoimmune diseases. It has been shown recently by Yin *et al.* that the two key metabolic pathways—glycolysis and mitochondrial oxidative metabolism—are elevated in cells from SLE patients as well as in mouse models of disease [96]. Using inhibitors of these pathways currently in the clinic—2-deoxy-D-glucose (2DG) and metformin—normalized T cell metabolism and decreased markers of SLE in animal models as well as in cells from SLE patients. These data suggest that inhibiting both glycolysis and mitochondrial metabolism could be a new therapeutic strategy for treating SLE. Similarly, it has been shown that PD-1 ligation alters T-cell metabolic reprogramming by inhibiting glycolysis and promoting lipolysis and fatty acid oxidation [97]. The study showed that PD-1 ligation promotes FAO of endogenous lipids by increasing expression of CPT1A, and inducing lipolysis as indicated by elevation of the lipase ATGL, the lipolysis marker glycerol and release of fatty acids. Conversely, CTLA-4 inhibits glycolysis without augmenting FAO, suggesting that CTLA-4 sustains the metabolic profile of non-activated cells. While FAO is believed to promote T cell memory, it is unclear why PD-1 expressing T cells exhibit an exhausted phenotype. Is it that both memory and exhausted T cells although rely on FAO, but have distinct metabolite signature that would result in differences in longevity and ability to mount a recall response? However, it must be noted that the PD-1 expression on T cells as exclusive marker of exhaustion has already been called in question by elegant studies where serial transfer of viral epitope specific PD-1 expressing T cells were shown to still control the magnitude of infection [98]. Additionally, blockade of PD-1 has been shown to decrease the efficacy of subsequent electron transport chain [99], and this metabolic inhibition need to be considered when using anti-PD-1 therapies in the clinic in order to augment long-term memory T cell responses.

Given that tumor cell themselves rely on the available glucose, it is likely that there is a competition between the effector T cells infiltrating the tumor. Some recent studies have elegantly shown that using the progressor or regressor tumors that it is not only the more progressor tumor that is highly glucolytic [100], but rapid depletion of available glucose by an aggressive tumor leads to a dysfunctional and exhausted T cell (as identified by enhanced expression of PD1). This study also showed that using antibodies against PD1 and CTLA4 – the T cells are better able to control tumors since these check-point blockade antibodies modulate the tumor metabolic commitment. Although, a question remains that if CTLA4 and PD1 act through different mechanisms [101], then do both pathways converge on the same metabolic pathway or a bifurcation point exits in the metabolism to account for the differences in mechanism of action.

One of the recent study has shown a new role for the glycolytic metabolite phosphoenolpyruvate (PEP) in sustaining T cell receptor-mediated Ca (2+)-NFAT signaling and effector functions by repressing sarco/ER Ca (2+)-ATPase (SERCA) activity. Tumor-specific CD4 and CD8 T cells could be metabolically reprogrammed by increasing PEP production through overexpression of phosphoenolpyruvate carboxykinase 1 (PCK1), which bolstered effector functions. Moreover, PCK1-overexpressing T cells restricted tumor growth and prolonged the survival of melanoma-bearing mice. Another study has recently shown that α-ketoglutarate (αKG),the glutamine-derived metabolite that enters into the mitochondrial citric acid cycle, acts as a metabolic regulator of CD4+ T cell differentiation [102]. This study showed that activation of naïve CD4+ T cells under conditions of glutamine deprivation resulted in their differentiation into Foxp3+ regulatory T (Treg) cells, which had suppressor function in vivo. Further, activation of glutamine-deprived naïve CD4+ T cells in the presence of a cell-permeableαKG analog increased the expression of the gene encoding the T-helper 1 (Th1) associated transcription factor T-bet and resulted in their differentiation into Th1 cells, concomitant with stimulation of mammalian target of rapamycin complex 1 (mTORC1) signaling. Thus, this paper established that a decrease αKG, caused intracellular by the amount of limited availability of extracellular glutamine, shifts the balance between the generation of Th1 and Treg cells toward that of a Treg phenotype. These above studies uncovers new metabolic checkpoints for T cell activity and demonstrates that metabolic reprogramming of tumor-reactive T cells can enhance anti-tumor T cell responses, illuminating new forms of immunotherapy.

Targeting glycolysis may not be always useful to control a T cell mediated disease state, since T cells activated in vivo by alloantigens in graft-versus-host disease (GVHD) increase mitochondrial oxygen consumption, fatty acid uptake, and oxidation, with small increases of glucose uptake and aerobic glycolysis. Using targeted metabolic 13C tracer fate association studies, to elucidate the metabolic pathway(s) employed by alloreactive T cells in vivo it was found that glutamine (Gln)-dependent tricarboxylic acid cycle anaplerosis is increased in alloreactive T cells and that Gln carbon contributes to ribose biosynthesis. This study further showed that pharmacological modulation of oxidative phosphorylation rapidly reduces anaplerosis in alloreactive T cells and improves GVHD [103]. Thus, T-cell metabolism is relevant to activated lymphocytes in vivo, with implications for the discovery of new drugs for immune disorders.

## CONCLUSION

Various studies with lymphoid and myeloid cells have highlighted the importance of understanding metabolism to design better approach for intervention in disease states. However, given the differences in metabolic fates between the lymphoid subsets (as T vs. NK vs. B) or myeloid cells (DC vs. MDSC vs. macrophages), the experimental strategies need to be carefully considered before reaching a definitive conclusion of the metabolic commitment since the dynamic metabolic states that depends upon activation state, activation signals, strength of activation etc. could result in differences in metabolism or metabolite accumulation that can lead to differences in function and viability of these cellular subtypes.
